# Increased perceived autonomy-supportive teaching in physical education classes changes students’ positive emotional perception compared to controlling teaching

**DOI:** 10.3389/fpsyg.2022.1015362

**Published:** 2022-11-01

**Authors:** Sascha Leisterer, Elias Paschold

**Affiliations:** Sport Psychology, Faculty of Sport Science, Leipzig University, Leipzig, Germany

**Keywords:** adolescents, didactics, exercise, fun, PE teacher behavior, school

## Abstract

Teachers can expect that autonomy support positively influences students’ affective-emotional perception in physical education (PE), when considering assumptions of the Self-Determination theory. Highly autonomy-supportive PE teaching comprises students’ free choices regarding organizational, procedural, and cognitive aspects of a PE lesson, whereas low autonomy support addresses these aspects only partly and controlling teaching refers to students as recipients of the teacher’s decisions. This quasi-experiment investigates effects to determine the effects of high autonomy-supportive (PE_high_), low autonomy-supportive (PE_low_) and controlling (PE_control_) PE class teaching styles on affective valence and enjoyment. As such, we compare the effects of these teaching styles on beneficial psychological outcomes (i.e., affective valence, enjoyment) in students. In a sample of German students (*N* = 57; age: *M* ± *SD* = 15.6 ± 0.6; gender: 53% female, 47% male) perceived autonomy support, affective valence, and enjoyment were assessed via self-report questionnaires before and after a 20-min PE class intervention focusing on high or low autonomy-supportive, or controlling teaching. Students who participated in PE_high_ perceived significantly more positive valence and enjoyment over time compared to students in the PE_low_ and PE_control_ groups (affective valence: *p* = 0.025, *η_p_^2^* = 0.13; enjoyment: *p* = 0.007, *η_p_^2^* = 0.17). Differences between groups show significant results for valence between PE_high_ and PE_control_, and between PE_low_ and PE_control_. Thus, PE_high_ should be preferred over PE_low_ to intensify these effects. Based on these results, PE teachers can employ a high autonomy-supportive teaching style (e.g., through a combination of free choices, social interaction, and informative feedback) to improve students’ positive affective-emotional perception and to foster an increase in students’ time engaged in physical activity.

## Introduction

Physical education (PE) teaching that focuses on implicit affective approaches, which are unspoken and oblivious teaching methods, to obtain adaptive student outcomes seems to be an effective and sustainable strategy to increase youth physical activity time (PA; Owen et al., 2014; Tilga et al., 2020; Oldervik and Lagestad, 2021). An implicit affective approach that is adopted to enhance students’ PA time both in PE and out of school is the use of an autonomy-supportive teaching style (Perez-Gonzalez et al., 2019). In the context of PE, an autonomy-supportive teaching style is expected to positively influence students’ both affective and emotional perception, which in turn is related to an increase in PA (Perez-Gonzalez et al., 2019; Zimmermann et al., 2021). In an effort to make a new contribution, we focus on the connection between students’ perception of autonomy-supportive PE teaching styles and the related affective-emotional outcomes.

According to the organismic-dialectical perspective of the Self-Determination theory (Deci and Ryan, 2000, 2013), motivation regulation processes can be described on a continuum reaching from external to internal motivation regulation. This continuum comprises external and introjected (i.e., external motivation regulation) and identified, integrated and intrinsic regulation (i.e., internal motivation regulation). Internal motivation regulation is seen as similar to the development of an internal locus of control by providing individual autonomy and freedom (Deci and Ryan, 2000). For example, internal motivation regulation can be observed in children during free play when they follow their inner desire to run, jump, move simply for enjoying themselves. Hence, being autonomous and free in one’s choices supports the individual in being responsible for her or his own outcomes (e.g., affective-emotional, behavioral) in life.

Within PE, several affective outcomes can be observed. Affective outcomes are individual perceptions, characterized by the two dimensions valence (i.e., feeling of pleasure-unpleasure) and arousal (i.e., feeling activated-deactivated) according to Russell (1980). These affects characterize subjective perceptions based on neurophysiological processes (Posner et al., 2005). As core affects, the perception of valence and arousal is essential for the development of emotions. For example, positive emotions have a positive valence with a more or less high intensity of arousal, and this is related to a tendency for approach behavior (Phaf et al., 2014). According to the Control-Value theory by Pekrun (2006), approaching an activity with an individual positive value (i.e., positive valence) and being totally in control of this activity (i.e., internal locus of control) can trigger the distinct emotion of enjoyment. Aligned to the organismic-dialectical perspective, this engagement in a personally important and freely chosen activity resembles to the concept of internal motivation regulation. Thus, it can be assumed that activities to improve internal motivation regulation might be connected to positive affective perception, which leads to enjoyment to this extent an internal locus of control can be perceived. These assumptions suggest that positive psychological outcomes can be expected in individuals when providing autonomy support, for example in educational contexts.

Within the context of PE, this means that the more students perceive autonomy support the more internally regulated (i.e., having an internal locus of control) their motivation will be. It may be assumed that students, who have acquired an internal locus of control regarding their learning process, by exploiting autonomy-supportive opportunities in PE, benefit from adaptive outcomes, for example, positive affective perception and emotions (Reeve et al., 2003; Leisterer and Jekauc, 2020). From a teacher’s perspective, autonomy support is a teacher-acquired behavior that focusses on supporting students’ ability to feel responsible for their own learning process, which represents the internal locus of control (Deci and Ryan, 2000; Reeve, 2015). In this responsibility-fostering approach, teachers assist students, for example, by providing free choices and alternatives, focusing on a democratic leadership style, giving instructional feedback, offering opportunities for social interaction, allowing time and space to learn, and respecting negative feelings through the learning process (Reeve and Jang, 2006; Reeve, 2015).

According to Stefanou et al. (2004), teachers may rely on organizational (i.e., autonomy in terms of time, space, social environment), procedural (i.e., autonomy in terms of creating the learning process), and cognitive (i.e., autonomy in terms of students’ cognitive reflection) autonomy-supportive teaching. Teachers can provide organizational autonomy support including opportunities to freely choose time for practice, group members, or space to practice; teachers can provide procedural autonomy support with free choice of media and material or decisions on how to evaluate performance; they can provide cognitive autonomy support with informational feedback, less teacher explanation and more student exploration, or problem-oriented tasks (Stefanou et al., 2004). Yet, to benefit from an autonomy-supportive PE teaching style, students must acknowledge these kinds of autonomy support.

To illustrate this, a PE class in which students can choose between different exercises to master a specific motor task is not autonomy-supportive *per se*, unless the teacher highlights how every exercise leads to mastery. Students who perceive autonomy support are likely to choose an appropriate exercise that suits their individual goal or according to their personal interest (i.e., cognitive autonomy support), by setting an individual time frame for mastering one exercise (i.e., organizational autonomy support), or by discussing with the teacher the best order of exercises to choose to ensure mastery (i.e., procedural autonomy support). Thus, while teachers can provide autonomy-supportive teaching styles–either organizational, procedural, or cognitive–the students also need to perceive these styles of autonomy support to develop an internal locus of control, leading to adaptive psychological outcomes (Deci and Ryan, 2000; Reeve et al., 2003).

Empirical research findings present a relationship between autonomy-supportive PE teaching and adaptive psychological outcomes for students (Perez-Gonzalez et al., 2019). First, autonomy support affects PA time (i.e., behavioral outcome) positively (Tilga et al., 2020). A review study highlights that autonomy-supportive PE teaching is a significant factor for improving students’ time engaged in PA (Owen et al., 2014), which can be explained by an increase of students’ internal locus of control (Moreno-Murcia et al., 2018). In contrast, controlling PE teaching hinders an internal locus of control and, consequently, students’ leisure time in PA decreases (Moreno-Murcia et al., 2018). Also, an autonomy-supportive teaching style creates opportunities to enhance students’ internal locus of control and to influence their affective-emotional perception (Behzadnia et al., 2018). Second, autonomy support in PE is related to students’ affective outcomes. A previous review study shows that autonomy-supportive PA influences students’ affective perception positively, whereas the question regarding the influence of different teaching styles and learning activities in PE on students’ affective perception remains open (Leisterer and Jekauc, 2020). Third, different approaches of autonomy-supportive teaching are associated with positive emotions in students. The review and meta-analysis by Rodríguez Macías et al. (2021) presents that non-traditional PE programs, which favor basic psychological need satisfaction, influence students’ perception of positive emotions significantly. Although specific mechanisms remain unexplained in this review and meta-analysis, perceived autonomy support–especially cognitive autonomy support–is associated with an increase in positive emotions (i.e., affective and emotional outcomes), which in turn are related to behavioral outcomes such as time engaged in PA (Lochbaum and Jean-Noel, 2016; Zimmermann et al., 2021). Thus far, it can be assumed that students who perceive autonomy support in PE are physically more active and have positive affective-emotional outcomes. However, the investigation of the effects of different teaching styles on beneficial psychological outcomes remains open in order to analyze underlying mechanisms between applied autonomy-supportive teaching styles and students’ perceptions.

Further investigation is needed of the mechanisms governing autonomy-supportive teaching styles, perceived autonomy support, and students’ affective-emotional outcomes in autonomy-supportive PE classes. In this way, we aim to uncover empirical evidence regarding the practical implications of different autonomy-supportive PE teaching styles and its adaptive effects on students’ affective-emotional outcomes. At present, there is an incomplete understanding of the influence of specific autonomy-supportive teaching styles (i.e., cognitive, organizational, procedural autonomy support) on students’ affects and emotions (Rodríguez Macías et al., 2021). To address this gap in the knowledge, we investigate the influences of different PE teaching styles on affective valence and emotions (i.e., enjoyment) in PE. In addition, we assess students’ perception of cognitive, organizational, and procedural autonomy support. With these results, we aim to advise PE teachers on what to focus on, when teaching in an autonomy-supportive manner to ensure a beneficial influence on students’ affective-emotional outcomes. Based on this aim, we ask the question regarding the connection that might exist between an autonomy-supportive PE teaching style, students’ autonomy support perception, their affective valence, and enjoyment. Accordingly, we set up the following hypotheses:

*H1*: Students who participate in a high autonomy-supportive PE class report higher levels of perceived autonomy support, affective valence, and enjoyment after their PE lesson compared to students in a low autonomy-supportive PE class.

*H2*: Students who participate in a high autonomy-supportive PE class report higher levels of perceived autonomy support, affective valence, and enjoyment after their PE lesson compared to students in a controlling PE class.

*H3*: Students who participate in a low autonomy-supportive PE class report higher levels of perceived autonomy support, affective valence, and enjoyment after their PE lesson compared to students in a controlling PE class.

## Materials and methods

A quasi-experimental design (for further information about quasi-experimental methodology, see Gopalan et al., 2020) with two measures was used to investigate within–between interactions affecting the connection of high autonomy-supportive (PE_high_), low autonomy-supportive (PE_low_), and controlling (PE_control_) PE class teaching styles on affective valence and enjoyment. This study was planned according to the American Psychological Association Ethical Principles of Psychologists and Code of Conduct (American Psychological Association, 2016) and the principles of the Declaration of Helsinki (World Medical Association, 2013). Prior to conducting this study, the authors’ institutional Ethics Advisory Board evaluated this study and reported its effects as harmless after reviewing the intervention procedure and its risks for the participants, principles of privacy and data security, participants’ informed consent, and participants’ right to withdraw without reasoning (Ethics Advisory Board evaluation number: 2021.06.02_eb_101).

### Sample

To calculate a required sample size for an ANCOVA for three intervention groups, G*Power (Faul et al., 2007) was used. Based on an assumed effect size of *f* = 0.60 (Lochbaum and Jean-Noel, 2016), *α* = 0.05, 1 − *β* = 0.95, and gender as a control variable, the calculation reveals a required sample size of *N* = 45 students. Two different secondary schools in Germany were asked to support this study. The schools accepted if PE teachers agreed on conducting the study in one of their PE lessons. Inclusion criteria were healthy PE students in either 9th or 10th grade (which represents students between 14 and 16 years of age), who were able to participate actively in PE. Additionally, only students with parental and individual consent to participate in this study were included. Consequently, three PE classes with *N* = 57 healthy and active students participated in this study as a convenient sample. All students and their parents gave their consent to participate in this study. No student of the three classes had to be excluded. Students were on average 15.6 years old with a standard deviation of 0.6; 53% of the participants reported to be female and 47% were male. One class was assigned to the high autonomy-supportive PE teaching intervention, whereby *n* = 17 students participated. A second class, consisting of *n* = 19 students, was assigned to the low autonomy-supportive PE teaching intervention. A third class with *n* = 21 students were assigned to the controlling PE class.

### Instruments and material

Self-report questionnaires assessed students’ perceived autonomy support, affective valence, and enjoyment in PE. In addition, age and gender were surveyed.

#### Perceived autonomy support

The German MD-PASS-PE (a full version of this questionnaire can be found here: Zimmermann et al., 2020) was used to assess the three factors, comprising organizational, procedural and cognitive autonomy support (e.g., organizational: “My PE teacher allows me to choose between different exercises”; procedural: “My PE teacher offers hints regarding how to do better”; cognitive: “My PE teacher is interested in what students want to do”). The 15 items of the questionnaire were responded according to 7-point Likert scale (ranging from “strongly disagree” to “strongly agree”), and, according to Zimmermann et al. (2020), had a good model fit for a bi-factorial model (i.e., model consisting of the three factors and a general factor); it also showed acceptable-to-good internal consistency reliability (Cronbach’s *α* = 0.72–0.81), and demonstrated criterion validity for intrinsic value and self-efficacy (Zimmermann et al., 2020).

#### Affective valence

The Self-Assessment Manikin (SAM; a full version of this questionnaire can be found here: Bradley and Lang, 1994) was used to measure students’ affective valence which is a discrete item, with a 9-point Likert scale from pleasure to unpleasure as a language-independent assessment tool. The SAM is reported as a highly reliable and valid questionnaire, as cross-correlations with similar assessment tools show: *r* = 0.98–0.99 (Bradley and Lang, 1994).

#### Enjoyment in PE

The German questionnaire FEFS-J (a full version of this questionnaire can be found here: Engels and Freund, 2019) assesses enjoyment in PE students regarding the three factors pleasure, flow, and relaxation (e.g., pleasure: “I have fun in PE”; flow: “Time flies by in PE”; relaxation: “PE increases my energy level for doing other things”). The FEFS-J consists of nine items with a 4-point Likert-scale ranging from “never” to “always.” The three-factor structure shows an acceptable model fit and the questionnaire’s internal consistency reliability is reported as acceptable to very good regarding subscales’ Cronbach’s *α* = 0.65–0.86 and total scales’ Cronbach’s *α* = 0.91 (Engels and Freund, 2019).

### Procedure

Students participated in the intervention and its assessment provided that their parents and the students themselves gave their consent after receiving information about the study 1 week prior to the intervention date. For data assessment, students were asked to create a pseudonym that they were supposed to write on every assessment questionnaire. All PE classes were given by the same teacher who was also the intervention supervisor (co-author).

**Figure 1:**
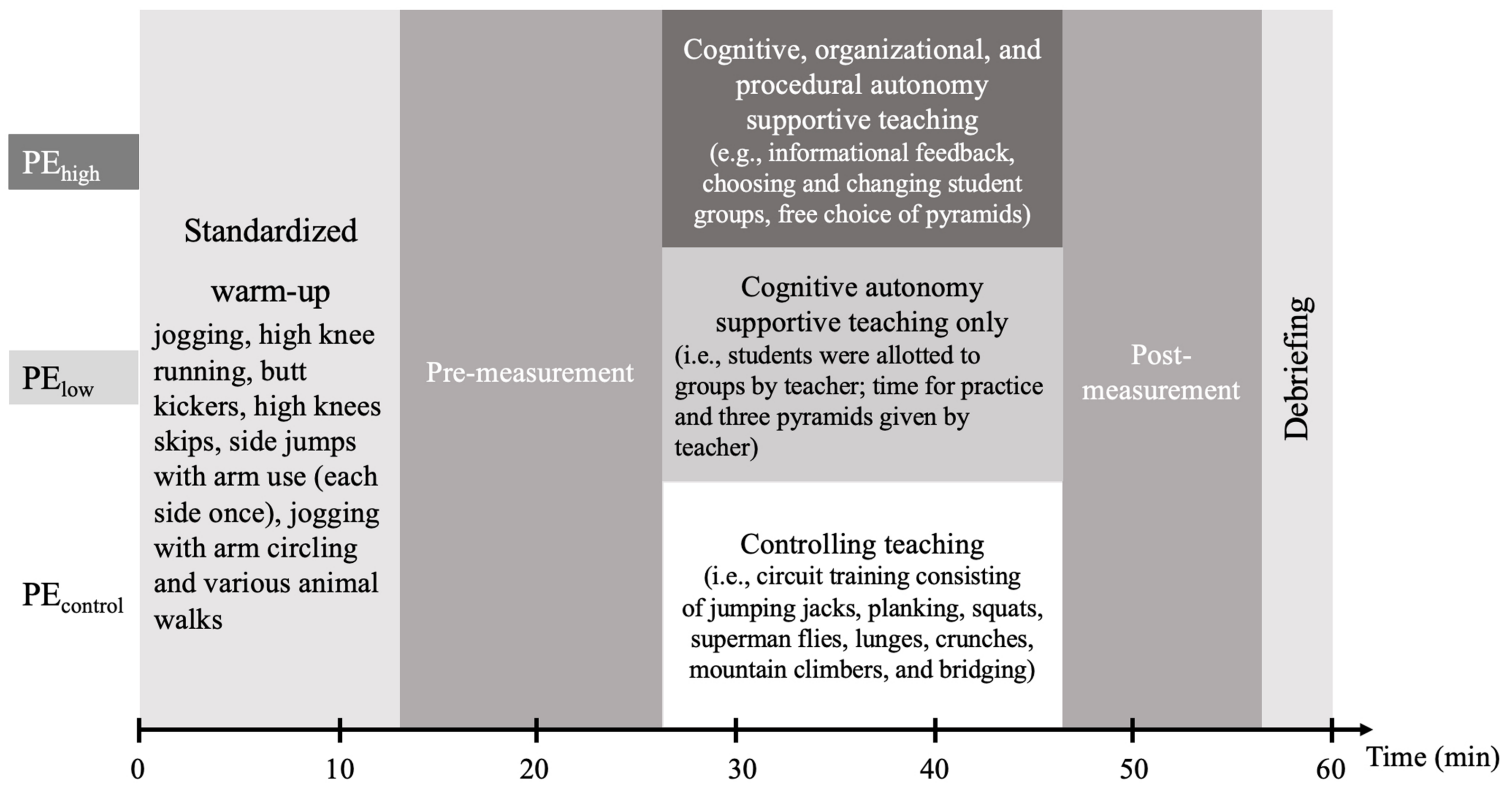
Experimental procedure; PE_high_, high autonomy-supportive PE teaching; PE_low_, low autonomy-supportive PE teaching; PE_control_, controlling PE teaching.

As depicted in Figure 1, the PE class started with a standardized introduction and warm-up before students were asked to answer the assessment questionnaires during pre-measurement. During the standardized warm-up, students had to cross the gym using one of the following exercises: jogging, high-knee running, butt kickers, high-knees skips, side jumps with arm use (each side once), jogging with arm circling and various animal walks. After the warm-up, the 20-min intervention phase started.

Based on the notions of organizational, procedural, and cognitive autonomy support (Stefanou et al., 2004), various types of autonomy support were provided in PE_high_ focusing on students’ choices following these manipulations:

- Offering cognitive autonomy support: students in PE_high_ could directly ask for feedback when needed; the teacher used non-controlling language (e.g., “Take your time. Normally, student groups practice about 5 min per pyramid. Check it out!”).
- Offering procedural autonomy support: students in PE_high_ could choose from 20 different human pyramid suggestions and were encouraged to create different groups when practicing.
- Offering cognitive and procedural autonomy support: the teacher provided a handout with human pyramid suggestions to support the students in their self-organized practice time.
- Offering organizational autonomy support: at first, the students practiced different gymnastics pyramids in small groups. Over time, we observed that students changed group compositions occasionally. In the last few minutes, one student suggested building a large gymnastics pyramid with the whole class. The class accepted the suggestion, and it was implemented after a short planning phase.

In PE_low_, the teaching style was limited to cognitive autonomy support to expect most impact on students’ psychological outcomes compared to organizational or procedural autonomy support (Lochbaum and Jean-Noel, 2016; Zimmermann et al., 2021). Therefore, and in contrast to PE_high_, these manipulations were realized by the teacher:

- Offering cognitive autonomy support: the teacher encouraged students to reflect and discuss within their group how to improve the creation of the human pyramid to provide cognitive autonomy support.
- Offering cognitive autonomy support: the teacher provided instructional feedback as in PE_high_, while the students were practicing.
- Limiting procedural autonomy support: the teacher demonstrated a human pyramid using one student group, before the other student groups were required to practice the particular pyramid for 5 min. No handout was provided and practice-time was controlled by the teacher.
- Limiting organizational autonomy support: the teacher allotted students to groups of four to five students, which could not be changed during practice.

In PE_control_, students depended on the teacher’s workout instructions. In comparison to PE_high_ and PE_low_, the objective of this intervention was not to learn how to build human pyramids but to practice physically to prepare for gymnastic activities in PE. Therefore, the following control situation was created:

- Limiting cognitive autonomy support: the teacher provided only corrective feedback when students did not execute exercises appropriately.
- Limiting procedural autonomy support: the teacher instructed students directly how to realize an exercise.
- Limiting organizational autonomy support: every student had his or her own mat to do a circuit training consisting of jumping jacks, planking, squats, superman flies, lunges, crunches, mountain climbers, and bridging.
- Limiting organizational autonomy support: students had no social interaction.

Following the intervention phase, students were asked again to fill out the assessment questionnaires post-measurement by using their pseudonyms.

### Data analysis

**Figure 2:**
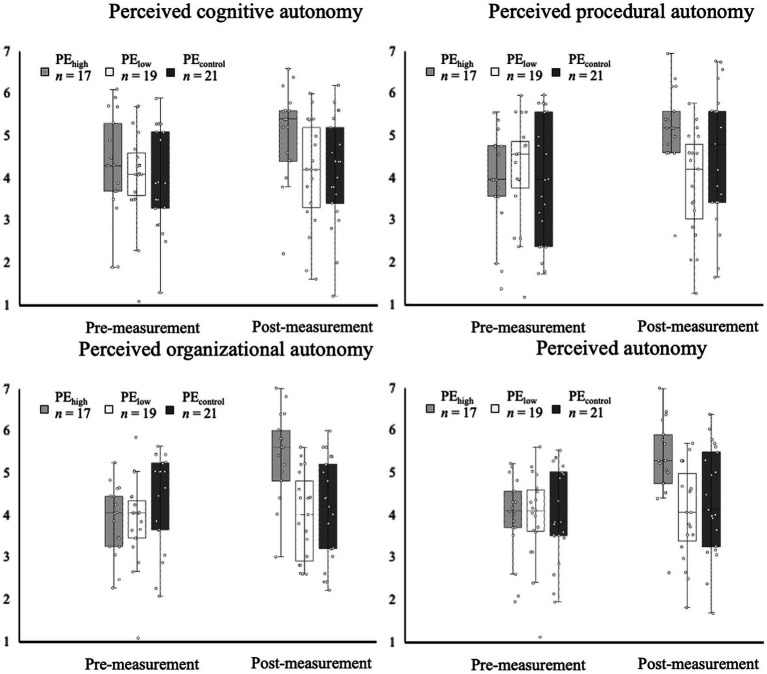
Boxplot charts show distribution of data points at pre- and post-measurement for perceived autonomy support—cognitive, procedural, organizational autonomy support, and total score of perceived autonomy support—in high (PE_high_), low autonomy-supportive (PE_low_) and controlling PE classes (PE_control_). Single data points provided.

**Figure 3:**
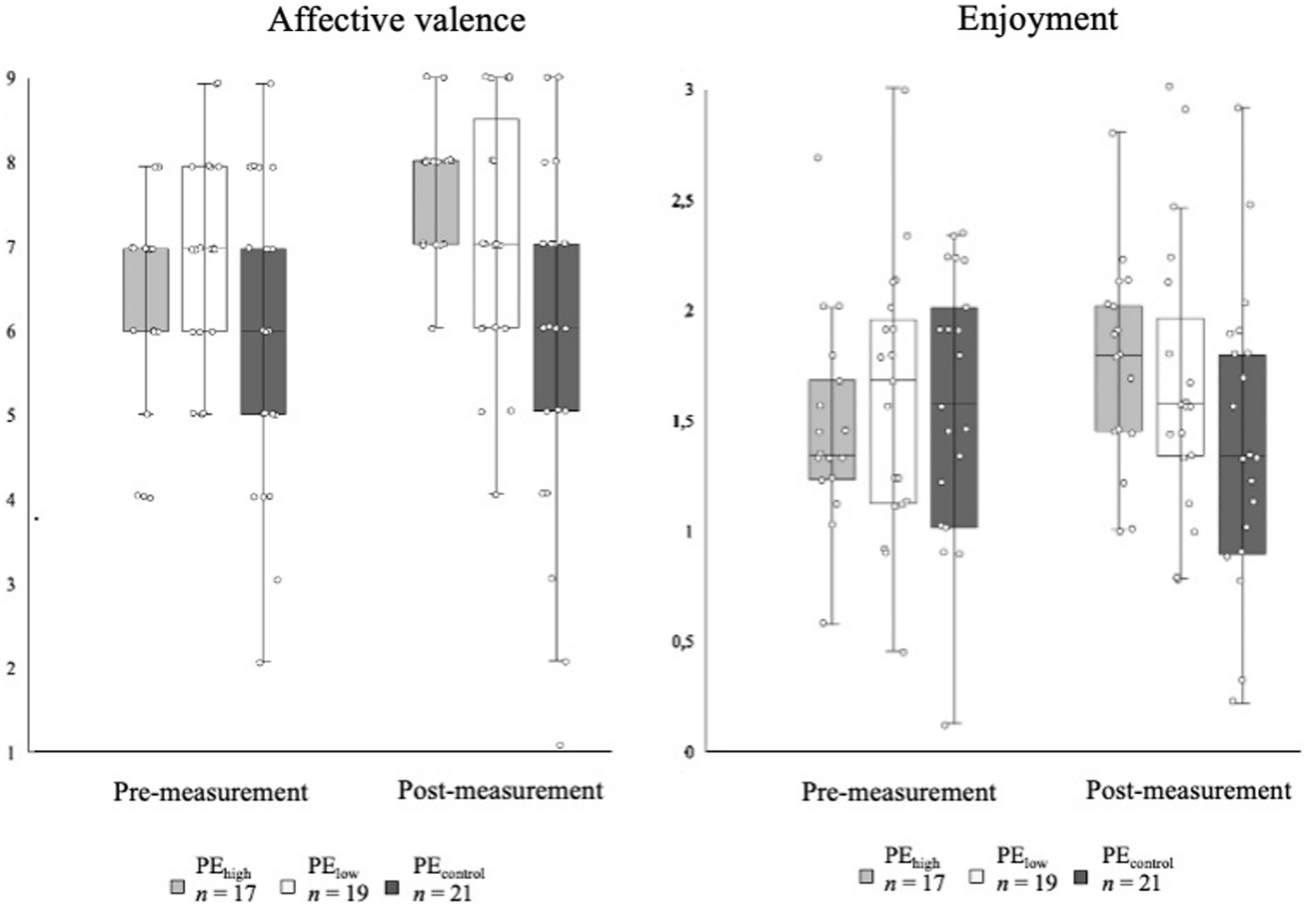
Outcome variables: Boxplot charts show distribution of data points at pre- and post-measurement for affective valence and enjoyment in high (PE_high_), low autonomy-supportive (PE_low_), and controlling PE classes (PE_control_). Single data points provided.

Data analysis was conducted in five steps using IBM SPSS statistics, version 27.0.1.0. First, data were prepared by matching all questionnaire scores to the students’ pseudonyms. The dataset was then checked for missing values. Second, descriptive statistics, outliers, and assumptions for hypotheses testing were checked. Outliers were found, however, these outliers are single data points of different individuals that may represent realistically the assessed sample and cannot be seen as systematic outliers. Thus, data were used as collected. The data fulfilled assumptions for further testing (independence of measurements, sphericity, normal distribution; distributions can be checked in Figures 2, 3). Referring to Blanca Mena et al. (2017) and Schmider et al. (2010), it can be assumed that subsequent analyses of variances are robust even for small sample sizes allotted to unequal intervention groups and non-normal distributed samples; thus, data was used as collected. Additionally, internal consistency of the used assessment tools were checked with this study’s sample. The MD-PASS-PE’s internal consistency of the three subscales (Cronbach’s *α*: cognitive autonomy support *α =* 0.83; procedural autonomy support *α* = 0.83; organizational autonomy support *α* = 0.73) and the total scale (Cronbach’s *α* = 0.91) can be reported as acceptable to very good. The internal consistency of the FEFS-J total scale can be reported as good (Cronbach’s *α* = 0.85). Third, one-way analyses of variances (ANOVA) were conducted with mean scores of the total score and subscore of perceived autonomy support to investigate statistically significant differences between the intervention groups at pre-measurement and to conduct a manipulation check. Fourth, to test our hypotheses, analyses of co-variances (ANCOVA) were conducted to analyze within-between interaction effects of perceived autonomy support, affective valence, and enjoyment over two measurements according to the intervention groups PE_high_, PE_low_, or PE_control_. Gender was included as a control variable. Fifth, Gabriel post-hoc tests were conducted to compare the effects between the three intervention groups.

## Results

### Descriptive statistics

**Figure 4:**
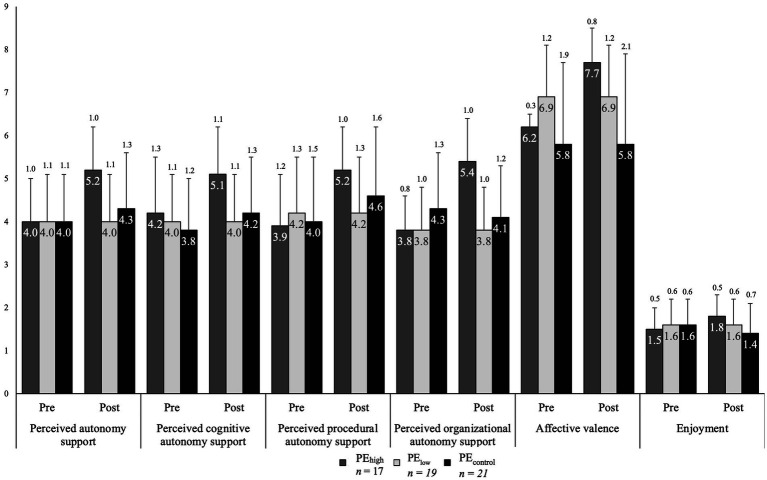
Descriptive mean comparisons (columns) with standard deviations (whiskers) of perceived autonomy support, cognitive autonomy, procedural autonomy, and organizational autonomy as well as affective valence and enjoyment in the three different PE classes (PE_high_, PE_low_, PE_control_) at pre- and post-measurement.

Perceived organizational, procedural, cognitive, and general autonomy support increased in every PE class, from pre- to post-measurement (Figure 4). However, in PE_low_ students reported a decrease in perceived procedural autonomy support and stably perceived general autonomy support from pre- to post-measurement. Also, students showed greatest increases in perceived autonomy support in all subscales of perceived autonomy support (i.e., organizational, procedural, cognitive) when participating in PE_high_. Descriptive statistics (see values in Figure 4) show that students in PE_high_ reported an increase in affective valence and enjoyment. Students in PE_control_ reported stable affective valence and a decrease in enjoyment despite an increase in perceived autonomy support.

### One-way ANOVA: Perceived autonomy support in subgroups

The three PE classes show no significant differences in perceived autonomy support (i.e., cognitive, procedural, organizational, and in general) at pre-measurement but at post-measurement both for the organizational, procedural, cognitive and the general autonomy support (see Table 1). Students in a PE_high_ perceive significantly more autonomy support than students in PE_low_ or PE_control_ [*F*(2, 56) = 5.94, *p* = 0.005, η_p_^2^ = 0.18]; students in PE_low_ and PE_control_ report no significant differences in their perception of autonomy support.

**Table 1:** Results of one-way ANOVA regarding perceived autonomy support (total score of the MD-PASS-PE) in the three different PE classes, pre- and post-measurement.

	*F*	*df1*	*df2*	*p*	η_p_^2^
**Perceived autonomy support**					
Pre-measurement	0.03	2	56	0.977	0.01
Post-measurement	5.94	2	56	0.005	0.18
**Perceived cognitive autonomy support**					
Pre-measurement	0.39	2	56	0.677	0.01
Post-measurement	3.55	2	56	0.036	0.12
**Perceived procedural autonomy support**					
Pre-measurement	0.27	2	56	0.767	0.01
Post-measurement	4.60	2	56	0.014	0.15
**Perceived organizational autonomy support**					
Pre-measurement	1.80	2	56	0.175	0.06
Post-measurement	8.91	2	56	< 0.001	0.25

### Within–between interaction effects

To test our hypothesis for within–between interaction effects of PE teachings styles on students’ perceived autonomy support, affective valence, and enjoyment, we performed analyses of co-variances (see Table 2 for all results) with gender as confounding variable and found significant effects for all outcome variables.

**Table 2:** Within–between interaction results of ANCOVA with two measurements for three PE subgroups (high and low autonomy-supportive, and controlling PE teaching) and the six variables of perceived autonomy support, affective valence, and enjoyment in PE.

	*F*	*df*	*p*	η_p_^2^
**Perceived autonomy support**				
Measurement	15.5	1	<0.001	0.23
Measurement × gender	0.81	2	0.373	0.02
Measurement × PE subgroup	13.71	2	<0.001	0.34
Error		53		
**Perceived cognitive autonomy support**				
Measurement	16.38	1	< 0.001	0.24
Measurement × gender	3.76	1	0.058	0.07
Measurement × PE subgroup	3.43	2	0.040	0.12
Error		53		
**Perceived procedural autonomy support**				
Measurement	9.60	1	0.003	0.15
Measurement × gender	0.56	1	0.459	0.01
Measurement × PE subgroup	10.92	2	< 0.001	0.29
Error		53		
**Perceived organizational autonomy support**				
Measurement	4.93	1	0.031	0.09
Measurement × gender	0.12	1	0.731	< 0.01
Measurement × PE subgroup	15.07	2	< 0.001	0.36
Error		53		
**Affective valence**				
Measurement	0.96	1	0.331	0.02
Measurement × gender	0.57	1	0.455	0.01
Measurement × PE subgroup	3.98	2	0.025	0.13
Error		53		
**Enjoyment in PE**				
Measurement	0.01	1	0.755	< 0.01
Measurement × gender	0.45	1	0.504	< 0.01
Measurement × PE subgroup	5.50	2	0.007	0.17
Error		53		

Gender as covariate. F, F-value; df, degrees of freedom; *p*, value of *p*; η_p_^2^, partial Eta-squared.

Perceived autonomy support increased in all three PE teaching style conditions during the intervention phase (Figure 2). In particular, PE_high_ students perceived highest levels of autonomy support in general as well as according to the organizational, procedural, and cognitive dimensions of perceived autonomy support (see Table 2). Only PE_low_ showed an opposite trend in perceived procedural autonomy support. Depicted in Figure 3, positive affective valence increases over time in PE_high_, whereas affective valence levels remain stable for PE_low_ and PE_control_. Regarding enjoyment (Figure 3), both PE_high_ and PE_low_ presented a positive development from pre- to post-measurement, whereas students in PE_control_ reported a decrease in enjoyment.

### *Post-hoc* tests

Subgroup differences in Gabriel post-hoc tests for PE teaching style revealed some significant differences (Table 3). At post-measurement, students in PE_high_ perceived significantly higher general autonomy support in comparison to PE_control_ students in (*ΔM* = 0.96, *SE* = 0.37, 95% CI [0.05; 1.87], *p* = 0.035). Regarding affective valence, students in PE_high_ and PE_low_ showed more positive affect compared to PE_control_, post-measurement (*ΔM* = 1.16, *SE* = 0.42, 95% CI [0.12; 2.20], *p* = 0.025; *ΔM* = 1.19, *SE* = 0.41, 95% CI [0.18; 2.20], *p* = 0.016, respectively). Comparisons regarding enjoyment showed no significant differences between the three different PE classes at post-measurement.

**Table 3:** Results of Gabriel *post-hoc*-comparison tests: Comparison of PE subgroup differences at post-measurement for the variables perceived autonomy support, affective valence, and enjoyment in PE.

	*ΔM*	*SE*	[CI_low_;CI_up_]	*p*
**Perceived autonomy support**				
PE_high_ vs. PE_low_	1.25	0.38	[0.32;2.18]	0.005
PE_low_ vs. PE_control_	−0.29	0.36	[−1.12;0.59]	0.806
PE_high_ vs. PE_control_	0.96	0.37	[0.05; 1.87]	0.035
**Perceived cognitive autonomy support**				
PE_high_ vs. PE_low_	0.57	0.37	[−0.34; 1.50]	0.335
PE_low_ vs. PE_control_	0.58	0.35	[−0.82; 0.93]	0.998
PE_high_ vs. PE_control_	0.64	0.37	[−0.26; 1.53]	0.238
**Perceived procedural autonomy support**				
PE_high_ vs. PE_low_	0.53	0.42	[−0.49; 1.55]	0.492
PE_low_ vs. PE_control_	−0.22	0.39	[−1.19; 0.75]	0.924
PE_high_ vs. PE_control_	0.31	0.41	[−0.68; 1.31]	0.823
**Perceived organizational autonomy support**				
PE_high_ vs. PE_low_	0.72	0.31	[−0.05;1.48]	0.072
PE_low_ vs. PE_control_	−0.33	0.29	[−1.10; 0.39]	0.600
PE_high_ vs. PE_control_	0.39	0.30	[−0.36; 1.13]	0.498
**Affective valence**				
PE_high_ vs. PE_low_	−0.03	0.43	[−1.10; 1.04]	1.00
PE_low_ vs. PE_control_	1.19	0.41	[0.18; 2.20]	0.016
PE_high_ vs. PE_control_	1.16	0.42	[0.12; 2.20]	0.025
**Enjoyment in PE**				
PE_high_ vs. PE_low_	−0.01	0.18	[−0.46; 0.44]	1.00
PE_low_ vs. PE_control_	0.15	0.17	[−0.27; 0.57]	0.769
PE_high_ vs. PE_control_	0.14	0.18	[−0.30; 0.57]	0.822

ΔM, mean difference; SE, standard error; CI_low_, 95% confidence interval, lower bound; CI_up_, 95% confidence interval, upper bound; *p*, value of *p*; PE_high_/_low_/_control_, high/low autonomy-supportive/controlling PE class.

## Discussion

### Autonomy-supportive PE teaching and its effects on students’ affects and enjoyment

This intervention study investigated autonomy-supportive PE teaching styles with regard to positive influences on students’ affects and emotions in PE, which in turn may be related to increases in PA and sport commitment. In this study, a high autonomy-supportive PE class applied organizational, procedural, and cognitive autonomy support, whereas a low autonomy-supportive PE class was restricted to cognitive autonomy-supportive teaching methods, and a controlling PE class was limited to a face-to-face teaching session with no autonomy support provided. First, we hypothesized that students who participated in a high autonomy-supportive PE class (i.e., PE_high_) will report higher levels of perceived autonomy support, affective valence and enjoyment after their PE lesson compared to students in a low autonomy-supportive PE class (i.e., PE_low_). Second, we hypothesized that students in PE_high_ will also report higher levels in the outcome variables compared to a controlling PE class (i.e., PE_control_). Third, we hypothesized that even students in PE_low_ achieve higher outcome values in autonomy support, affective valence, and enjoyment than students in PE_control_.

Regarding perceived autonomy support, the three different PE teaching styles support different levels of perceived autonomy support as assumed: a highly autonomy-supportive PE teaching style is perceived as such, whereas in lower autonomy-supportive or controlling PE teaching lower autonomy support is perceived. Yet, in PE classes which provided low autonomy support (i.e., cognitive autonomy support only), students perceived less procedural autonomy support since they lacked choices regarding task, materials, or evaluation which would be needed to perceive procedural autonomy. Surprisingly, students perceived slightly more autonomy support in a controlling PE teaching class than in a low autonomy-supportive class. Thus, when focusing on perceived autonomy support, the first and second hypothesis can be accepted but the third has to be rejected.

With regard to affective valence and enjoyment, all three alternative hypotheses can be accepted with some limitations: a high autonomy-supportive teaching style has the strongest effect on students’ positive affect and enjoyment compared to low autonomy-supportive or controlling PE teaching, based on the statistically significant interaction effects. However, when comparing the differences between PE_high_, PE_low_, and PE_control_ after the intervention, statistically significant results can be found only for affective valence, highlighting that PE_high_ achieves better effects than PE_low_ in comparison to a controlling teaching style. These significant results are lacking for enjoyment despite significant interaction effects. In sum, it can be assumed that even a short high autonomy-supportive PE teaching lesson has a substantial influence on students’ perceived autonomy support and affective valence, whereas low autonomy support shows effects especially on students’ positive affect.

With regard to adaptive psychological outcomes, students participating in high and low autonomy-supportive PE teaching classes reported a positive influence of affective valence compared to a controlling PE teaching style, whereas a high autonomy-supportive teaching style seems to have a larger effect, which is in line with recent assumptions (Perez-Gonzalez et al., 2019; Leisterer and Jekauc, 2020). Autonomy-supportive teaching styles have a beneficial impact on students’ enjoyment but, in the present study, reveal no significant difference compared to a controlling teaching style. This finding refers to Zimmermann et al. (2021) that perceived cognitive and procedural autonomy support in PE classes correlate positively with enjoyment, when assessed for a longer time period. Referring to Pekrun (2006), it can be assumed that a short intervention–as in the present study–cannot lead to an increase in distinct emotions, such as enjoyment, despite an increase in positive affective perception. Presumably, students might need more time to feel in control of the task, in other words to develop an internal locus of control. Regarding the controlling teaching style, no influence on students’ affective valence can be observed, although it slightly reduces students’ enjoyment. No observed effect on affective valence may show that PE_control_ students were not frustrated with regard to perceived autonomy support, because they might have an existing internal locus of control. As recently shown by Behzadnia et al. (2018), the lack of an internal locus of control (i.e., frustration of the need to perceive autonomy support) seems to have an impact on affective valence. In addition to Rodríguez Macías et al. (2021), the present findings point out that an autonomy-supportive PE teaching style can be assumed as an effectful intervention to satisfy students’ need for autonomy and, by that, have an impact on their affective perception. However, future studies should assume that students’ internal locus of control is a relevant factor in the perception of autonomy support, even when controlling PE teaching styles are used.

In a nutshell, this study contributes to filling the gap of knowledge about the effects of different PE teaching styles on students’ affective-emotional outcomes (Leisterer and Jekauc, 2020; Rodríguez Macías et al., 2021). Autonomy-supportive PE teaching shows strong effects on the development of affective valence and enjoyment. Thus, teachers should favor autonomy-supportive teaching over controlling teaching to influence students’ positive affective valence. In particular, the present results suggest that teachers could address different aspects of autonomy-supportive teaching (i.e., organizational, procedural, cognitive) at the same time, and not focus on only one aspect of autonomy support. This means that in practice, PE teachers should apply autonomy-supportive PE teaching as a complex of cognitive, procedural, and organizational autonomy support simultaneously, to ensure students’ positive affect and enjoyment, which are crucial for a commitment to PA and sports (Tilga et al., 2020). Consequently, we can conclude that autonomy-supportive teaching is an effective implicit instructional approach for PE teachers to assist students in developing a physically active lifestyle.

As shown in previous studies, positive affect and enjoyment in PE classes contribute to PA in youth (Lochbaum and Jean-Noel, 2016; Behzadnia et al., 2018; Moreno-Murcia et al., 2018; Perez-Gonzalez et al., 2019; Tilga et al., 2020; Zimmermann et al., 2021). Based on our findings, and, in line with current literature, PE teachers could provide high autonomy-supportive teaching (i.e., providing cognitive, procedural, and organizational autonomy support) to positively influence students’ affects and emotions *via* perceived autonomy support (Moreno-Murcia et al., 2018). For example, students’ affective-emotional perception in PE class may be affected by providing students the opportunity to explore their own way of moving (i.e., cognitive autonomy support) with freely chosen material (i.e., procedural autonomy support) in a setting where students are allowed to choose time and space for their practice (i.e., organizational autonomy support; Tilga et al., 2020; Zimmermann et al., 2021). Moreover, we have shown that cognitive, procedural, and organizational autonomy-supportive teaching influences students’ perception of autonomy support, which is connected to positive affect and enjoyment.

Despite these positive findings, we have neglected the dark side of Self-Determination theory. Following the work of Trigueros et al. (2019), contrasting autonomy frustration (i.e., lack of possibilities to satisfy the need for autonomy) in controlling PE classes with autonomy-supportive teaching might be a future branch of experimental research in PE classes. Exploring autonomy frustration in controlling PE, will provide a better understanding of the influence of different teaching styles on students’ psychological outcomes related to time spent in PA. Finally, we can assume that the relationship between autonomy support and affective-emotional outcomes also supports students’ PA time according to recent literature (Lochbaum and Jean-Noel, 2016; Zimmermann et al., 2021). Future research should focus on investigating the effects of different autonomy-supportive teaching styles (i.e., organizational compared to procedural compared to cognitive autonomy support) on students’ adaptive outcomes. Lastly, PE teachers might learn to be more autonomy-supportive in vocational education; here, future research could also investigate the psychological effects on educators when teaching PE classes in an autonomy-supportive manner (Tilga et al., 2021).

### Practical implications

This study reveals that students benefit psychologically, when PE teachers create their classes considering organizational, procedural, and cognitive autonomy-supportive, all at once. To implement this complex repertoire of autonomy support, teachers have to learn different ways of being autonomy-supportive in PE classes. Therefore, current knowledge regarding autonomy-supportive teaching must be disseminated, especially to schools and teachers, for example through teacher education. Vocational workshops could be offered for expert and novice teachers alike, and teacher students could learn autonomy-supportive teaching methods at university. At the beginning, it might be recommended to learn how to apply one way of autonomy support and to work for the complex of organizational, procedural, and cognitive autonomy support step-by-step. For example, teachers and teacher students might benefit from focusing on being organizational autonomy-supportive, first, by asking their students for their favorite place to practice or by offering students free time to practice. Then, PE teachers could add procedural autonomy-supportive teaching methods, such as discussing with students evaluation goals, and cognitive autonomy-supportive teaching methods, such as giving informative feedback. Applying high autonomy-supportive teaching in PE has the potential to improve students’ psychological outcomes, which in turn supports their time spent in PA.

### Limitations

This study is quasi-experimental. Although its ecological validity is high and the investigated classes can benefit from the results, its internal validity is limited due to the lack of an experimental control (e.g., sample randomization). This generalization issue can be addressed by more investigations with randomized samples. In addition, a randomized sample and balanced group sizes should be taken into account in order to reduce the risk of potential violation of assumptions in future studies. Also, the content of the interventions (i.e., building human pyramids, fitness workout) might have influenced the outcome variables, which should be considered in future studies, for example, by controlling for students’ perceived physical exertion. Nevertheless, the intervention treatments in this study were standardized (e.g., intervention protocol, test supervisor equals teacher) to detect the best effect of autonomy-supportive teaching on affective-emotional perception. One teacher for all three intervention groups is both disadvantageous and advantageous. The risk of Pygmalion effects (Rosenthal, 1973) might rise with one teacher as responsible for all interventions; nevertheless, the benefit in this study was that the teacher strictly realized the intervention protocol, which controlled secondary variances, such as teacher personality or gender, to improve internal validity of this quasi-experiment. Consequently, this means that the PE class was partially alienated from an original PE class. In addition, 20-min interventions were very short. For longitudinal effects, experiments with longer and repetitive interventions with multiple measurements should be conducted. Since the associations between positive affect, enjoyment and youth PA are already well described, this study did not assess the type of motivation, PA time in youth, nor students’ locus of control. Thus, future studies should focus on investigating autonomy-supportive teaching in original PE class settings over several weeks, assessing PA time spent both in school and in leisure activities, as well as investigating students’ locus of control. Furthermore, effects of autonomy-supportive teaching on teachers themselves, such as the benefits that PE educators experience when teaching autonomy-supportively, need to be examined further. To address this question, future studies might also assess teachers’ psychological outcomes when teaching PE autonomy-supportively.

## Conclusion

Students’ affective-emotional perception depends on their perception of autonomy-supportive teaching in PE. This quasi-experimental study with three different PE classes shows that high autonomy-supportive teaching has strongest effects on students’ positive affect and enjoyment. Similar effects of low autonomy-supportive and controlling PE teaching depend on students’ perception of autonomy support, and might be more likely when students have an internal locus of control. These findings highlight the importance of adopting an all-encompassing autonomy-supportive teaching approach in PE, favoring organizational, procedural, and cognitive autonomy support. Thus, employing high autonomy-supportive PE teaching can improve students’ affective-emotional perception in PE and could increase students’ PA time.

## Data availability statement

The raw data supporting the conclusions of this article will be made available by the authors, without undue reservation.

## Ethics statement

The studies involving human participants were reviewed and approved by Leipzig University, Ethics Advisory Board evaluation number: 2021.06.02_eb_101. Written informed consent to participate in this study was provided by the participants’ legal guardian/next of kin.

## Author contributions

SL: idea, conceptualization, data analysis, and first draft writing. SL and EP: methodological planning and revising of the manuscript. EP: intervention and data collection. All authors contributed to the article and approved the submitted version.

## Funding

The authors acknowledge support from the German Research Foundation (DFG) and Universität Leipzig within the program of Open Access Publishing.

## Conflict of interest

The authors declare that the research was conducted in the absence of any commercial or financial relationships that could be construed as a potential conflict of interest.

## Publisher’s note

All claims expressed in this article are solely those of the authors and do not necessarily represent those of their affiliated organizations, or those of the publisher, the editors and the reviewers. Any product that may be evaluated in this article, or claim that may be made by its manufacturer, is not guaranteed or endorsed by the publisher.
